# Identification of Virulence Genes and Antibiotic Resistance in Extraintestinal Pathogenic *Escherichia coli* Isolated from Broiler Carcasses Using MALDI-TOF MS

**DOI:** 10.3390/pathogens14050501

**Published:** 2025-05-20

**Authors:** Jia-Tong Han, Yu-Xuan Tang, Si-Yi Wu, Yi-Ran Chen, Zhan-Peng Zou, Hang Zeng, Zhongjia Yu

**Affiliations:** 1 School of Animal Science and Technology, Foshan University, Foshan 528225, China; hy98322@gmail.com (J.-T.H.); m18054244233@163.com (Y.-X.T.); wsy1319225030@163.com (S.-Y.W.); 18870276121@163.com (Y.-R.C.); 15217385208@163.com (Z.-P.Z.); 2 School of Food and Bioengineering, Xihua University, Chengdu 610039, China; zenghang@mail.xhu.edu.cn; 3 Key Laboratory of Food Microbiology of Sichuan, Xihua University, Chengdu 610039, China

**Keywords:** chicken carcasses, MALDI-TOF MS, extraintestinal pathogenic *Escherichia coli* (ExPEC), multidrug resistance, virulence genes

## Abstract

*Escherichia coli* contamination in poultry is a significant concern due to its potential to cause foodborne illness. The presence of extraintestinal pathogenic *E. coli* (ExPEC) strains in chicken carcasses can lead to severe human infections. This study investigates the prevalence, virulence, and antibiotic resistance of *E. coli* isolates from chicken carcasses processed in both wet market and industrial environments, with a focus on the detection capabilities of MALDI-TOF MS. A total of 119 *E. coli* isolates were obtained. Only a small proportion (5/119) carried enteropathogenic virulence genes. In contrast, 71.42% (85/119) of the isolates harbored multiple extraintestinal virulence genes. Among these, *iucC* and *sitA*, which are associated with systemic infections, were present in 68.24% (58/85) and 43.53% (37/85) of the isolates, respectively. Furthermore, 47.06% (56/119) of the isolates carrying at least two extraintestinal virulence genes were classified as ExPEC. Additionally, 94.6% (54/56) of ExPEC isolates were multidrug resistant (MDR), showing resistance to over three antibiotic classes, raising concerns about the spread of antibiotic resistance. MALDI-TOF MS profiling revealed significant heterogeneity among the ExPEC isolates, with no distinct clustering patterns based on processing environment or sampling site. These findings underscore the public health risks posed by ExPEC in poultry and emphasize the need for improved surveillance, stringent hygiene practices, and responsible antibiotic use in poultry production.

## 1. Introduction

Extraintestinal pathogenic *Escherichia coli* (ExPEC) is a significant public health concern due to its ability to cause a wide range of extraintestinal infections in humans and animals. Unlike commensal *E. coli* strains such as *Proteus mirabilis* that inhabit the gut without causing harm, ExPEC possesses an array of virulence factors that enable it to colonize, invade, and damage host tissues, leading to infections such as urinary tract infections (UTIs), neonatal meningitis, and septicemia [1]. In poultry, avian pathogenic *E. coli* (APEC), a subset of extraintestinal pathogenic *E. coli* (ExPEC), causes colibacillosis in birds [2]. APEC may contaminate broiler carcasses during processing; however, the impact of environmental factors remains unclear [3]. Broiler carcasses have been recognized as a potential reservoir for various pathobionts, including enterotoxigenic *E. coli* (ETEC), *Proteus mirabilis*, ExPEC, and other opportunistic pathogens, raising concerns about their role in zoonotic transmission and foodborne infections [4,5]. The presence of these pathogens and pathobionts in broiler carcasses presents a serious risk to consumers, as contamination during processing and handling can lead to the spread of virulent and antimicrobial-resistant strains [6,7]. Unlike ETEC, which carries well-characterized enteropathogenic virulence genes such as *fliC*, *stx1*, *stx2*, *eae*, *rfbE*, and *hlyA*, the presence of virulence genes in ExPEC isolated from broiler carcasses remains poorly characterized [1,8,9]. Moreover, compared to industrial processing, slaughtering at wet markets, which is commonly practiced in many Asian countries, has a higher likelihood of spreading virulent strains to consumers [4]. However, there are few comparative studies analyzing the presence of ExPEC between broiler carcasses processed at wet markets and those processed industrially [3,10].

The increasing prevalence of antimicrobial resistance (AMR) among ExPEC strains isolated from food-producing animals further exacerbates the threat posed by these pathogens [11]. Although legislation aimed at limiting antibiotic use in food animal production has helped to control antibiotic resistance, the use of antibiotics in poultry production has contributed to the emergence and spread of multidrug-resistant (MDR) strains. These strains pose a significant public health concern, as they can compromise the effectiveness of commonly used antibiotics in human medicine. Resistance genes carried by ExPEC may be transferred to other bacterial populations through horizontal gene transfer, such as via plasmids, further amplifying the challenge of controlling bacterial infections [12]. Understanding the virulence and resistance profiles of ExPEC strains from broiler carcasses is therefore essential for developing effective control strategies and ensuring food safety.

To accurately identify and characterize ExPEC strains, advanced and reliable diagnostic methods are required. Traditional bacterial identification techniques, such as culture-based methods and biochemical assays, are often time-consuming and may lack precision in distinguishing pathogenic strains from commensal ones. In recent years, matrix-assisted laser desorption/ionization time-of-flight mass spectrometry (MALDI-TOF MS) has emerged as a powerful tool for bacterial identification, offering rapid, accurate, and cost-effective characterization of microbial species [13]. MALDI-TOF MS allows for precise protein profiling of bacterial isolates, enabling differentiation between closely related strains. This technology has seen increasing application in clinical and food microbiology settings, where it is used to enhance the identification of pathogens and support epidemiological investigations [14,15,16]. Despite the growing use of MALDI-TOF MS in microbiological research, there are still limited data on its application in identifying ExPEC from poultry sources.

The present study aimed to identify extraintestinal pathogenic genes in *E. coli* isolated from broiler carcasses to determine the prevalence and foodborne risk of ExPEC. Additionally, the peptide mass fingerprint (PMF) of these isolates was collected using MALDI-TOF MS and correlated with virulence genes and antibiotic resistance profiles to explore potential associations between PMF patterns and virulence or resistance determinants. The present study contributes to a broader understanding of ExPEC epidemiology in poultry and highlights the necessity of improved pathogen surveillance, stricter biosecurity measures, and more stringent antibiotic regulations in the poultry industry to mitigate public health risks.

## 2. Materials and Methods

### 2.1. Bacterial Isolates and Identification

A total of 119 *E. coli* isolates were obtained using *E. coli* Chromogenic Medium (HB7001, Haibo, Qingdao, China) from Foshan, China (23°1′ N 113°6′ E) in a previous study [4]. Briefly, 21 chicken carcasses were purchased randomly from 11 different wet markets and 12 carcasses were purchased from 6 different supermarkets. After purification on tryptic soy agar (TSA, 028074, Huankai, Guangzhou, China), each suspected isolate was checked using polymerase chain reaction (PCR) targeting the *E. coli*-specific *16S rRNA* gene and further identified using matrix-assisted laser desorption/ionization time-of-flight mass spectrometry (MALDI-TOF MS).

Genomic DNA was extracted using an alkaline lysis method [17]. Briefly, a single colony was suspended in 20 µL of lysis buffer (2.5 mL of 10% SDS (BS088, Baisha, Beijing, China), 5 mL of 1N NaOH (1010310101700, XiLong, Shantou, China), and 92.5 mL of Milli-Q water) and heated at 95 °C for 15 min. After brief centrifugation, 180 µL of Milli-Q water was added, followed by a 5 min centrifugation at 10,000× *g* at 4 °C. The *E. coli*-specific 16S rRNA gene fragment (585 bp) was amplified using the primers and annealing conditions listed in Supplementary Table S1. The PCR (final volume: 25 µL) contained 2 µL of template DNA, 0.5 µL of each primer (10 µM), 12.5 µL Fast PCR Master Mix (RR350A, Takara, Osaka, Japan), and 9.5 µL of Milli-Q water. PCR was performed with an initial denaturation at 94 °C for 5 min, followed by 30 cycles of denaturation at 94 °C for 1 min, annealing at 55 °C for 1 min, and extension at 72 °C for 1 min, with a final extension at 72 °C for 6 min, and the amplicon size was confirmed by electrophoresis on a 1.2% agarose gel (BY-R0100, Biowest, Nuaillé, France).

For MALDI-TOF MS identification (refer to a previous study [16]), a single colony from TSA was picked using a sterile toothpick and smeared onto a MALDI-TOF MS target plate (Bruker Daltonics, Bremen, Germany). After air-drying, the sample was overlaid with 1 µL of a matrix solution containing 10 mg/mL α-cyano-4-hydroxycinnamic acid (8201344, Bruker, Bremen, Germany) in acetonitrile (A298777, Aladdin, Shanghai, China), deionized water, and trifluoroacetic acid (767449, Macklin, Shanghai, China) with 50:47.5:2.5 (v/v/v). Calibration was performed using a bacterial test standard (BTS 155 255343; Bruker Daltonics) before each run. Mass spectra were generated using a Microflex LT MALDI-TOF mass spectrometer (Bruker Daltonics) operating in linear positive ion detection mode under Bruker flexControl 3.4 software (Bremen, Germany). Species identification was achieved by comparing mass spectra against the Bruker MSP database (version DB5989) using Bruker Compass 5.0 software (Bremen, Germany), with scores ≥ 2.0 considered as confident species-level identifications.

### 2.2. Virulence Gene Detection

DNA from confirmed *E. coli* isolates was subjected to multiplex PCR for detecting enteropathogenic virulence genes, including *fliC, stx1, stx2, eae, rfbE,* and *hlyA,* as per previously established protocols [8]. The primer sequences and expected amplicon sizes are provided in Supplementary Table S1. PCR conditions involved 30 cycles, and amplicons were visualized on a 1.2% agarose gel.

For isolates that tested negative for enteropathogenic virulence genes, additional PCR screening was performed to detect extraintestinal virulence genes, including *sfa*, *iss*, *tsh*, *kpsmtII*, *kpsmtK1, iucC, hlyD, papC, ibeA,* and *sitA*. The primer sequences and annealing conditions are listed in Supplementary Table S1. While *ibeA* and *sitA* were assessed using multiplex PCR, all other genes were analyzed using single-gene PCR under similar cycling conditions. Amplicon sizes were verified via electrophoresis on a 1.2% agarose gel.

### 2.3. Antibiotic Resistance Determination

Isolates carrying two or more extraintestinal virulence genes were classified as ExPEC, as defined in a previous study [18]. These isolates were subjected to antimicrobial susceptibility testing using the disk diffusion method according to Clinical and Laboratory Standards Institute (CLSI) M100 guidelines [19]. The following antibiotics, commonly used in human medicine and veterinary practice, were tested: aminoglycosides: amikacin (AMK, 30 µg, 240827J701,Yinuokang, Nanjing, China), gentamicin (GEN, 10 µg, 40827J707, Yinuokang); β-lactams: ampicillin (AMP, 10 µg, 240827J102, Yinuokang), aztreonam (AZI, 15 µg, 240827J804, Yinuokang), piperacillin (PIP, 100 µg, 40827J107, Yinuokang), amoxicillin–clavulanic acid (AMC, 20/10 µg, 240827J201,Yinuokang), sulbactam–ampicillin (SAM, 10/10 µg, 240827J202, Yinuokang), tazobactam–piperacillin (TZP, 110 µg, 240827J301, Yinuokang); first-generation cephalosporins: cefazolin (CZ, 30 µg, 240827J523, Yinuokang); third- or fourth-generation cephalosporins: cefepime (FEP, 30 µg, 240827J503, Yinuokang), cefotaxime (CTX, 30 µg, 240827J519,Yinuokang), ceftazidime (CAZ, 30 µg, 240827J520, Yinuokang); carbapenems: imipenem (IPM, 10 µg, 240910J603, Yinuokang), meropenem (MEM, 10 µg, 240827J602, Yinuokang); quinolones: ciprofloxacin (CIP, 5 µg, 240827J904, Yinuokang), levofloxacin (LEV, 5 µg, 240827J914, Yinuokang), moxifloxacin (MXF, 5 µg, 240827J907, Yinuokang); and others: chloramphenicol (C, 30 µg, 240827J1902, Yinuokang), tetracycline (TE, 30 µg, 240827J1003, Yinuokang), and sulfamethoxazole–trimethoprim (SXT, 1.23/23.75 µg, 240827J1501, Yinuokang). Antibiotic susceptibility was determined based on CLSI M100 breakpoints, and the results were interpreted accordingly.

### 2.4. Data Analysis

Mass spectrum files generated from MALDI-TOF MS were converted to mzXML format using MSConvert 3.0.25064 software [20]. Dendrogram clustering was performed using IDBac, according to a previously established approach [21]. Clustering was conducted separately based on peak presence and mass intensity, including peaks with ≥70% similarity. Different mass cutoff pairs were applied for comparison. The distance algorithm was set to cosine, and the clustering algorithm was set to the unweighted pair group method with arithmetic mean (UPGMA). The resulting Newick format dendrograms were visualized using ImageGP [22], incorporating metadata on virulence genes and antibiotic resistance profiles.

## 3. Results

### 3.1. Determination of ExPEC in Chicken Carcasses Processed in Different Environments

According to a previous study, a total of 91.3% (21/23) of broilers slaughtered at wet markets and 100% (12/12) of those processed in industrial facilities tested positive for *Escherichia coli*, yielding a total of 119 isolates [4]. Among these, 68.91% (82/119) isolates were obtained from 21 broilers slaughtered at wet markets, while 45.12% (37/119) were recovered from 12 carcasses processed in industrial settings. Based on sampling site distribution, 33 isolates were identified from neck skin, 38 from breast skin, and 48 from back skin.

**Figure 1:**
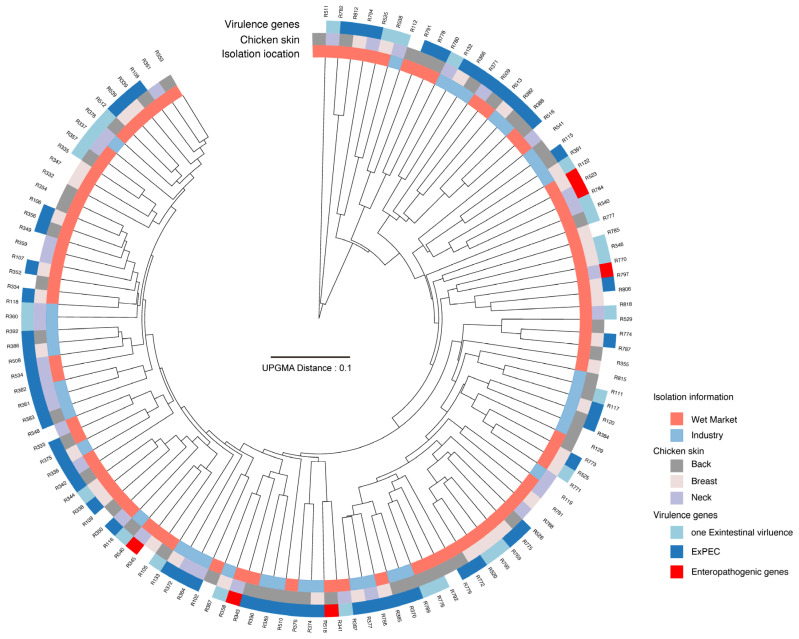
Dendrogram of 119 *E. coli* isolates, showing isolation information and associated virulence genes. The dendrogram clustered *E. coli* strains based on their PMFs from MALDI-TOF MS.

**Figure 2:**
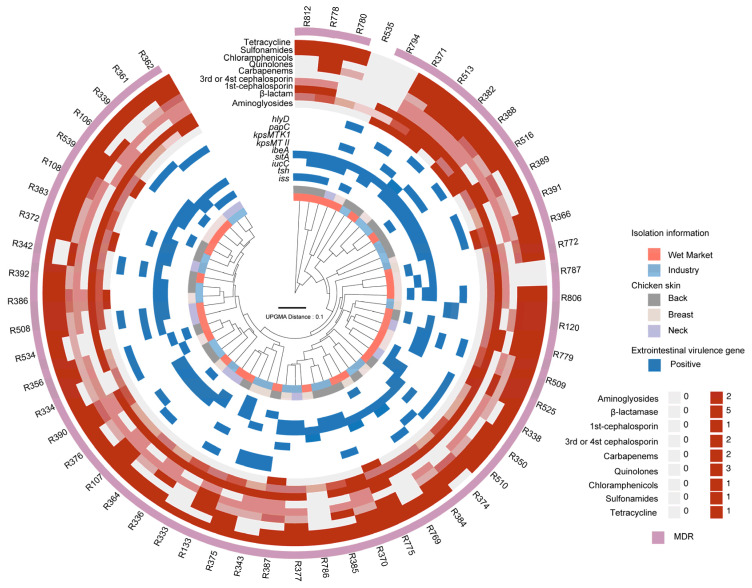
Dendrogram of 56 EXPEC isolates showing isolation information, associated extraintestinal virulence genes, and antibiotic resistance profiles. The dendrogram is based on the protein mass fingerprints (PMFs) generated by MALDI-TOF MS.

Among the 119 *E. coli* isolates, six carried enteropathogenic virulence genes, including three with *fliC*, one with *eae*, one with *stx1*, and one harboring both *fliC* and *stx1*. Apart from the 35 isolates in which no virulence genes were detected, 85 isolates carried extraintestinal virulence genes, as illustrated in Figure 1. Specifically, 1.18% (1/85) of isolates carried *sfa*, 21.18% (18/85) contained *iss*, 11.76% (10/85) harbored *tsh*, 43.53% (37/85) carried *sitA*, 68.24% (58/85) carried *iucC*, 5.88% (5/85) contained *ibeA*, 15.29% (13/85) harbored *kpsMT II*, 1.18% (1/85) contained *kpsMT K1*, 11.76% (10/85) carried *papC*, and 35.29% (30/85) harbored *hlyD*. A total of 56 (65.88%) isolates carrying at least two virulence genes were classified as extraintestinal pathogenic *E. coli* (ExPEC), as depicted in Figure 1. Among these, 17.86% (10/56) isolates carried four virulence genes, 35.71% (20/56) harbored three, and 48.21% (27/56) contained two (see Figure 2).

Among the 56 ExPEC isolates, 32 were recovered from 76.19% (16/21) of broiler carcasses slaughtered at wet markets, while 24 were obtained from 66.7% (8/12) of carcasses processed in industrial facilities. Furthermore, 19.64% (11/56) isolates were obtained from neck skin, 37.50% (21/56) from breast skin, and 42.86% (24/56) from back skin.

### 3.2. Multidrug Resistance in ExPEC Isolates

As presented in Table 1 and Figure 2, antibiotic resistance was observed among ExPEC isolates derived from broiler carcasses. Specifically, 19.64% (11/56) of strains were resistant to aminoglycosides, 98.21% (55/56) were resistant to β-lactams, and 92.86% (52/56) were resistant to cephalosporins, with resistance rates of 92.86% (52/56) to first-generation cephalosporins and 41.07% (23/56) to third- and fourth-generation cephalosporins. Additionally, 73.21% (41/56) of isolates exhibited resistance to carbapenems, 83.93% (47/56) to quinolones, 58.93% (33/56) to chloramphenicol, 92.86% (52/56) to tetracycline, and 87.5% (49/56) to sulfamethoxazole–trimethoprim. In addition, several isolates possessed intermediate resistance to these antibiotics, of which 19.64% (11/56) were intermediate to sulbactam–ampicillin, ceftazidime and chloramphenicol; 21.43% (12/56) to amoxicillin–clavulanic acid and ciprofloxacin; 23.21% (13/56) to cefepime; and 51.89% (29/56) to levofloxacin, suggesting extensive potential for antibiotic resistance threatening food safety.

Notably, only 10.71% (6/56) of ExPEC isolates were sensitive to more than 15 antibiotic agents, whereas 53.57% (30/56) demonstrated resistance to over 50% of the 20 antibiotics tested (Table 2). Three isolates exhibited resistance to more than 14 antibiotic agents and carried multiple virulence genes, as R363 carried *iss*, *iucC* and *papC*; R382 carried *iucC*, *sitA*, *ibeA*, and *hlyD*; and R513 carried *sitA* and *kpsMT II*. According to the classification criteria for multidrug-resistant (MDR) strains [23], which define MDR as resistance to more than three distinct antibiotic classes, 94.6% (54/56) of ExPEC isolates were classified as MDR.

### 3.3. PMF Analysis Reveals Heterogeneity in Virulence and Antibiotic Resistance Profiles

Using different mass cutoffs and clustering parameters based on intensity and peak presence, no distinct clustering patterns were observed among isolates (Supplementary Figure S1). With regard to virulence profiles, isolates carrying enteropathogenic virulence genes, extraintestinal virulence genes, and those lacking virulence determinants were not well clustered.

As illustrated in Figure 1, isolates from different processing environments, sampling sites, and virulence backgrounds did not exhibit a distinct clustering pattern, indicating high heterogeneity in the MALDI-TOF MS protein mass fingerprinting (PMF) profiles of *E. coli* isolates. Despite some ExPEC isolates forming clusters in Figure 1, additional PMF clustering was conducted based on specific virulence genes across different mass cutoffs and parameters (see Supplementary Figure S2). Notably, isolates carrying *sitA* and *iucC*, which were prevalent among the sampled isolates, exhibited a more discernible clustering pattern. A mass cutoff between 5000 and 12,000 produced better clustering, and this cutoff was used to generate Figure 2, which provides an overview of the clustering information. However, the antibiotic resistance profiles of ExPEC isolates did not exhibit a clear pattern in relation to PMF clustering or virulence profiles, further underscoring the substantial heterogeneity in isolation background, virulence gene composition, and antibiotic resistance characteristics of ExPEC isolates.

## 4. Discussion

The findings of the present study highlight the high prevalence of *E. coli* in chicken carcasses processed in both wet market and industrial environments, with a significant proportion of isolates classified as ExPEC (47.06%). A very low rate of *E. coli* isolates carrying enteropathogenic virulence genes (*fliC, stx1, stx2, eae, rfbE,* and *hlyA*) was detected. This may be attributed to food safety policies that include clear standards for controlling enteropathogenic *E. coli*, such as *E. coli* O157:H7 (e.g., GB 29921-2021) [24]. However, the presence of *E. coli* strains harboring multiple extraintestinal virulence genes indicates a potential risk for human infections, highlighting the need for stringent hygiene measures during poultry processing.

There was no distinct prevalence of ExPEC between carcasses from wet markets (76.19%) and industrial facilities (66.7%), suggesting that the source of contamination likely originates from the broilers themselves—such as their intestinal content, feathers, and feces—rather than from the processing environment. The reservoir of ExPEC/APEC remains a topic of debate, particularly regarding whether it stems from chicken microbiota or another source [25]. The present study supports the hypothesis that ExPEC may originate from chicken microbiota, as ExPEC was detected across different processing environments and sampling sites, exhibiting high heterogeneity. In addition to the gut microbiota, the lung microbiota also serves as a reservoir for ExPEC, as it naturally colonizes the lungs of chickens [26]. Chicken products are believed to be a source of ExPEC transmission from animals to humans. Both the present study and previous studies have observed high heterogeneity among ExPEC isolates from chicken-derived products [5,27].

The total number of isolates in this study was relatively small, with only 119 isolates from 21 carcasses at wet market and 12 at industry, yet 47.06% were classified as ExPEC. This proportion is significantly higher than that reported in previous studies, where only 2.4% of isolates from healthy chickens in China and 21% from chicken meat in the USA were identified as ExPEC [3,5]. A key difference between the present study and previous ones is the current use of single PCR to identify a subset of extraintestinal virulence genes, which likely increased the detection rate. Furthermore, the widespread presence of ExPEC in broiler carcasses suggests that the risk of this opportunistic pathogen has been underestimated.

The risk associated with foodborne ExPEC is not only due to the presence of virulence genes, but also its antibiotic resistance. In this study, 94.6% of ExPEC isolates were resistant to more than three categories of antibiotics, classifying them as multidrug-resistant (MDR) strains. Among these, isolates resistant to more than 14 antimicrobial agents carried virulence genes associated with systemic infections, such as *iucC* and *sitA* [28]. Additionally, *papC* and *hlyD*, which assist in ExPEC invasion [29], were also found in these pan-resistant isolates. Although the expression of these virulence genes was not confirmed through animal experiments, the high level of antimicrobial resistance—94.6% of ExPEC isolates identified as multidrug-resistant (MDR)—suggests a potentially overlooked risk associated with chicken carcasses. Since 2013, regulations on antibiotic use in animals have been increasingly strengthened in China, aiming to control the spread of antibiotic-resistant bacteria originating from animals [30,31]. However, in the year of the present study, ExPEC isolates from poultry markets still exhibited multiple antibiotic resistance, suggesting that the natural purification process requires a longer period to achieve significant reduction.

The present study aimed to evaluate the potential of protein mass fingerprinting (PMF) generated by MALDI-TOF MS for the rapid identification of ExPEC (extraintestinal pathogenic *Escherichia coli*) or antibiotic-resistant strains. However, clustering analysis revealed significant heterogeneity among ExPEC isolates regarding their virulence and antibiotic resistance profiles. Although some clustering was observed within specific mass ranges, no distinct patterns emerged based on processing environments or sampling sites. This finding suggests that ExPEC strains exhibit substantial genetic and phenotypic diversity, complicating efforts to develop standardized detection and control strategies.

Moreover, the lack of correlation between virulence genes and antibiotic resistance profiles supports the notion that ExPEC isolates evolve through diverse mechanisms, potentially acquiring resistance independently of virulence traits. MALDI-TOF MS has been established as a fast and reliable tool for bacterial identification and has also been applied to screen for resistance and virulence biomarkers [16,32,33], such as those used for detecting ESCAPE [34]. This technology is considered a promising approach for the identification of antibiotic resistance in foodborne pathogens [35]. However, the failure to achieve clear clustering in the PMF patterns of ExPEC highlights the need for further efforts to explore specific biomarkers of virulence and resistance using MALDI-TOF MS.

In conclusion, the present study provides critical insights into the prevalence, virulence, and antibiotic resistance of ExPEC isolates from chicken carcasses. The high rates of MDR strains highlight the urgent need for improved surveillance, strict biosecurity measures, and responsible antibiotic use in poultry production to mitigate the public health risks associated with ExPEC.

## Figures and Tables

**Table 1:** Antibiotic-resistant ExPEC counts.

Classes	Antimicrobial Agents	Susceptible	Intermediate	Resistant	Resistant for the Class
Aminoglycosides	AMK	51	1	4	11/56
	GEN	39	8	9	
β-lactams	AMC	17	12	27	55/56
	AMP	6	2	48	
	AZI	27	7	22	
	PIP	47	7	2	
	SAM	31	11	14	
	TZP	2	1	53	
Cephalosporins	CZ	4	0	52	52/56
	FEP	41	13	2	
	CTX	34	3	19	
	CAZ	40	11	5	
Carbapenems	IPM	13	2	41	41/56
	MEM	51	3	2	
Quinolones	CIP	21	12	23	47/56
	LEV	12	29	15	
	MXF	8	3	45	
Others	C	12	11	33	33/56
	TE	2	2	52	52/56
	SXT	7	1	48	49/56

**Table 2:** MDR ExPEC strains counts.

Resistance		ExPEC Counts
≤5 agents (5/20)		10.71% (6/56)
≥10 agents (10/20)		53.7% (30/56)
≥14 agents (14/20)		5.56% (3/56)
MDR	≥3 classes (8)	94.6% (54/56)

## Data Availability

The raw data supporting the conclusions of this article will be made available by the authors on request.

## Supplementary Materials

The following supporting information can be downloaded at https://www.mdpi.com/article/10.3390/pathogens14050501/s1. Figure S1: Dendrogram of 119 Escherichia coli isolates with associated virulence genes, generated based on protein mass fingerprints (PMFs) from MALDI-TOF MS with varying mass cutoffs and clustering parameters; Figure S2: Dendrogram of 56 Escherichia coli isolates with associated extraintestinal virulence genes, generated based on protein mass fingerprints (PMFs) from MALDI-TOF MS with varying mass cutoffs and clustering parameters; Table S1: Primers information. References [36,37,38] are cited in the supplementary materials.

## References

[1] Jakobsen L., Spangholm D.J., Pedersen K., Jensen L.B., Emborg H.-D., Agersø Y., Aarestrup F.M., Hammerum A.M., Frimodt-Møller N. (2010). Broiler Chickens, Broiler Chicken Meat, Pigs and Pork as Sources of ExPEC Related Virulence Genes and Resistance in *Escherichia coli* Isolates from Community-Dwelling Humans and UTI Patients. Int. J. Food Microbiol..

[2] Jamali H., Akrami F., Bouakkaz S., Dozois C.M. (2024). Prevalence of Specific Serogroups, Antibiotic Resistance and Virulence Factors of Avian Pathogenic *Escherichia coli* (APEC) Isolated from Clinical Cases: A Systematic Review and Meta-Analysis. Microb. Pathog..

[3] Zou M., Ma P.-P., Liu W.-S., Liang X., Li X.-Y., Li Y.-Z., Liu B.-T. (2021). Prevalence and Antibiotic Resistance Characteristics of Extraintestinal Pathogenic *Escherichia coli* among Healthy Chickens from Farms and Live Poultry Markets in China. Animals.

[4] Zhou Y., Wang N., Cen J., Han J., Tang Y., Xu Z., Zeng H., Houf K., Yu Z. (2025). Comparative Analyses of Bacterial Contamination and Microbiome of Broiler Carcasses in Wet Market and Industrial Processing Environments. Int. J. Food Microbiol..

[5] Mitchell N.M., Johnson J.R., Johnston B., Curtiss R., Mellata M. (2015). Zoonotic Potential of *Escherichia coli* Isolates from Retail Chicken Meat Products and Eggs. Appl. Environ. Microbiol..

[6] Lamar F., Mondlane-Milisse A., Brito D.R.A., Mucache H.N., Jesser K.J., Fagnant-Sperati C.S., Victor C., Shioda K., Fafetine J.M., Saíde J.Â.O. (2025). Accumulation of Microbial Hazards and Assessment of Food Hygiene Associated with Broiler Chicken Processing at Open Air Food Markets in Maputo, Mozambique. Int. J. Food Microbiol..

[7] Elshebrawy H.A., Kasem N.G., Sallam K.I. (2025). Methicillin- and Vancomycin-Resistant *Staphylococcus aureus* in Chicken Carcasses, Ready-to-Eat Chicken Meat Sandwiches, and Buffalo Milk. Int. J. Food Microbiol..

[8] Sanches M.S., Baptista A.A.S., de Souza M., Menck-Costa M.F., Koga V.L., Kobayashi R.K.T., Rocha S.P.D. (2019). Genotypic and Phenotypic Profiles of Virulence Factors and Antimicrobial Resistance of *Proteus mirabilis* Isolated from Chicken Carcasses: Potential Zoonotic Risk. Braz. J. Microbiol..

[9] Pavlickova S., Klancnik A., Dolezalova M., Mozina S.S., Holko I. (2017). Antibiotic Resistance, Virulence Factors and Biofilm Formation Ability in *Escherichia coli* Strains Isolated from Chicken Meat and Wildlife in the Czech Republic. J. Environ. Sci. Health Part B Pestic. Food Contam. Agric. Wastes.

[10] Gharaibeh M.H., Al Sheyab S.Y., Malkawi I.M., Al Qudsi F.R. (2024). Phenotypic and Genotypic Characterization of *Escherichia coli* Isolated from the Chicken Liver in Relation to Slaughterhouse Conditions. Heliyon.

[11] Pitout J. (2012). Extraintestinal Pathogenic *Escherichia coli*: A Combination of Virulence with Antibiotic Resistance. Front. Microbiol..

[12] Koskella B., Hall L.J., Metcalf C.J.E. (2017). The Microbiome beyond the Horizon of Ecological and Evolutionary Theory. Nat. Ecol. Evol..

[13] Yu Z., Joossens M., Houf K. (2020). Analyses of the Bacterial Contamination on Belgian Broiler Carcasses at Retail Level. Front. Microbiol..

[14] Sumbana J.J., Santona A., Fiamma M., Taviani E., Deligios M., Zimba T., Lucas G., Sacarlal J., Rubino S., Paglietti B. (2021). Extraintestinal Pathogenic *Escherichia coli* ST405 Isolate Coharboring *BlaNDM-5* and *BlaCTXM-15*: A New Threat in Mozambique. Microb. Drug Resist..

[15] Wozniak-Biel A., Bugla-Płoskońska G., Kielsznia A., Korzekwa K., Tobiasz A., Korzeniowska-Kowal A., Wieliczko A. (2018). High Prevalence of Resistance to Fluoroquinolones and Tetracycline *Campylobacter* Spp. Isolated from Poultry in Poland. Microb. Drug Resist..

[16] Yu Z.J., Peruzy M.F., Dumolin C., Joossens M., Houf K., Yu Z., Peruzy M.F., Dumolin C., Joossens M., Houf K. (2019). Assessment of Food Microbiological Indicators Applied on Poultry Carcasses by Culture Combined MALDI-TOF MS Identification and *16S rRNA* Amplicon Sequencing. Food Microbiol..

[17] Edwards U., Rogall T., Blöcker H., Emde M., Böttger E.C. (1989). Isolation and Direct Complete Nucleotide Determination of Entire Genes. Characterization of a Gene Coding for 16S Ribosomal RNA. Nucleic Acids Res..

[18] Johnson J.R., Murray A.C., Gajewski A., Sullivan M., Snippes P., Kuskowski M.A., Smith K.E. (2003). Isolation and Molecular Characterization of Nalidixic Acid-Resistant Extraintestinal Pathogenic *Escherichia coli* from Retail Chicken Products. Antimicrob. Agents Chemother..

[19] Weinstein M.P. (2020). Performance Standards for Antimicrobial Susceptibility Testing; Twenty-Fifth Informational Supplement.

[20] Chambers M.C., Maclean B., Burke R., Amodei D., Ruderman D.L., Neumann S., Gatto L., Fischer B., Pratt B., Egertson J. (2012). A Cross-Platform Toolkit for Mass Spectrometry and Proteomics. Nat. Biotechnol..

[21] Clark C.M., Costa M.S., Sanchez L.M., Murphy B.T. (2018). Coupling MALDI-TOF Mass Spectrometry Protein and Specialized Metabolite Analyses to Rapidly Discriminate Bacterial Function. Proc. Natl. Acad. Sci. USA.

[22] Chen T., Liu Y.-X., Huang L. (2022). Image GP: An Easy-to-Use Data Visualization Web Server for Scientific Researchers. iMeta.

[23] Nikaido H. (2009). Multidrug Resistance in Bacteria. Annu. Rev. Biochem..

[24] (2021). National Food Safety Standards Limits of Pathogenic Bacteria in Prepackaged Foods.

[25] Riley L.W. (2020). Distinguishing Pathovars from Nonpathovars: *Escherichia coli*. Microbiol. Spectr..

[26] Antão E.-M., Glodde S., Li G., Sharifi R., Homeier T., Laturnus C., Diehl I., Bethe A., Philipp H.-C., Preisinger R. (2008). The Chicken as a Natural Model for Extraintestinal Infections Caused by Avian Pathogenic *Escherichia coli* (APEC). Microb. Pathog..

[27] Lu Q., Zhang W., Luo L., Wang H., Shao H., Zhang T., Luo Q. (2022). Genetic Diversity and Multidrug Resistance of Phylogenic Groups B2 and D in InPEC and ExPEC Isolated from Chickens in Central China. BMC Microbiol..

[28] Li D., Reid C.J., Kudinha T., Jarocki V.M., Djordjevic S.P. (2020). Genomic Analysis of Trimethoprim Resistant Extraintestinal Pathogenic *Escherichia coli* (ExPEC) and Recurrent Urinary Tract Infections. Microb. Genom..

[29] Harwalkar A., Gupta S., Rao A., Srinivasa H. (2014). Lower Prevalence of *HlyD*, *PapC* and *Cnf-1* Genes in Ciprofloxacin-Resistant Uropathogenic *Escherichia coli* than Their Susceptible Counterparts Isolated from Southern India. J. Infect. Public Health.

[30] Zhao Q., Jiang Z., Li T., Cheng M., Sun H., Cui M., Zhang C., Xu S., Wang H., Wu C. (2023). Current Status and Trends in Antimicrobial Use in Food Animals in China, 2018–2020. One Health Adv..

[31] Shao Y., Wang Y., Yuan Y., Xie Y. (2021). A Systematic Review on Antibiotics Misuse in Livestock and Aquaculture and Regulation Implications in China. Sci. Total Environ..

[32] Ashfaq M.Y., Da’na D.A., Al-Ghouti M.A. (2022). Application of MALDI-TOF MS for Identification of Environmental Bacteria: A Review. J. Environ. Manag..

[33] Yoon E.-J., Jeong S.H. (2021). MALDI-TOF Mass Spectrometry Technology as a Tool for the Rapid Diagnosis of Antimicrobial Resistance in Bacteria. Antibiotics.

[34] Flores-Treviño S., Garza-González E., Mendoza-Olazarán S., Morfín-Otero R., Camacho-Ortiz A., Rodríguez-Noriega E., Martínez-Meléndez A., Bocanegra-Ibarias P. (2019). Screening of Biomarkers of Drug Resistance or Virulence in ESCAPE Pathogens by MALDI-TOF Mass Spectrometry. Sci. Rep..

[35] Feucherolles M., Cauchie H.-M., Penny C. (2019). MALDI-TOF Mass Spectrometry and Specific Biomarkers: Potential New Key for Swift Identification of Antimicrobial Resistance in Foodborne Pathogens. Microorganisms.

[36] Parvin M.S., Talukder S., Ali M.Y., Chowdhury E.H., Rahman M.T., Islam M.T. (2020). Antimicrobial Resistance Pattern of *Escherichia coli* Isolated from Frozen Chicken Meat in Bangladesh. Pathogens.

[37] Bai J., Shi X., Nagaraja T.G. (2010). A Multiplex PCR Procedure for the Detection of Six Major Virulence Genes in *Escherichia coli* O157:H7. J. Microbiol. Methods.

[38] Xia X., Meng J., Zhao S., Bodeis-Jones S., Gaines S.A., Ayers S.L., McDermott P.F. (2011). Identification and Antimicrobial Resistance of Extraintestinal Pathogenic *Escherichia coli* from Retail Meats. J. Food Prot..

