# Supplementary material for: Identification of Virulence Genes and Antibiotic Resistance in Extraintestinal Pathogenic Escherichia coli Isolated from Broiler Carcasses Using MALDI-TOF MS
Source: Pathogens. 2025 May 20;14(5):501. doi: 10.3390/pathogens14050501 (PMC12114469; doi:10.3390/pathogens14050501)
Supplement: Supplementary file 1 [file pathogens-14-00501-s001.zip › pathogens-3580987-supplementary.pdf]

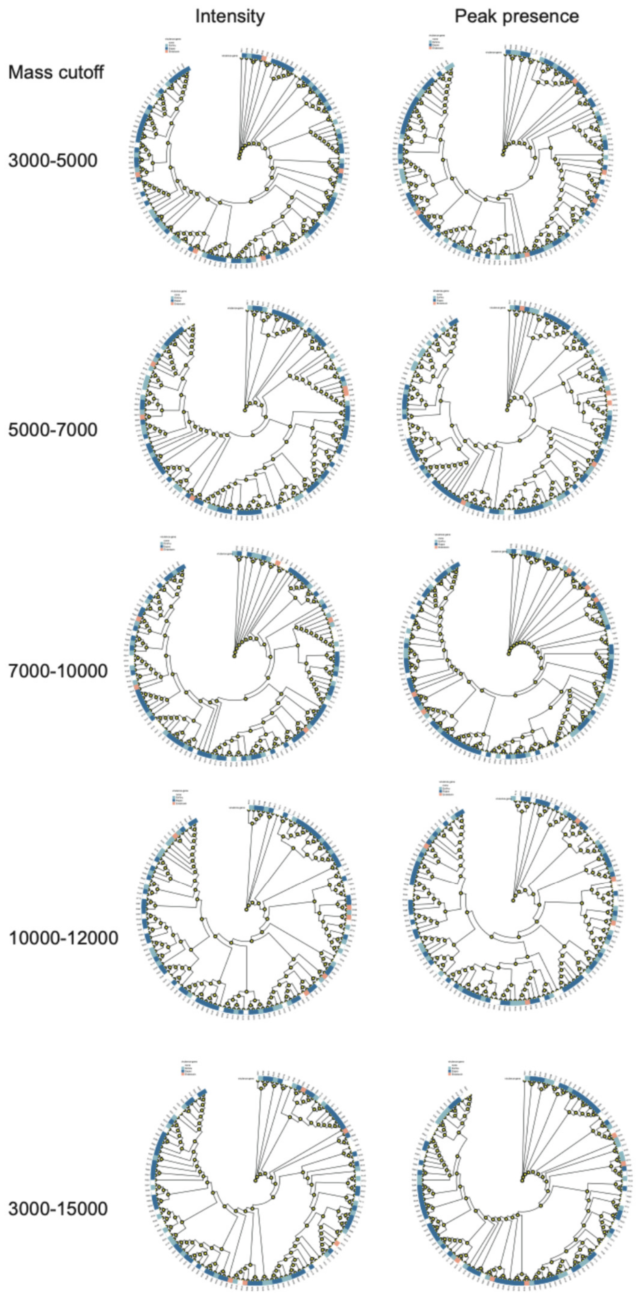

Figure S1. Dendrogram of 119 Escherichia coli isolates with associated virulence genes, generated based on protein mass fingerprints (PMFs) from MALDI-TOF MS with varying mass cutoffs and clustering parameters.

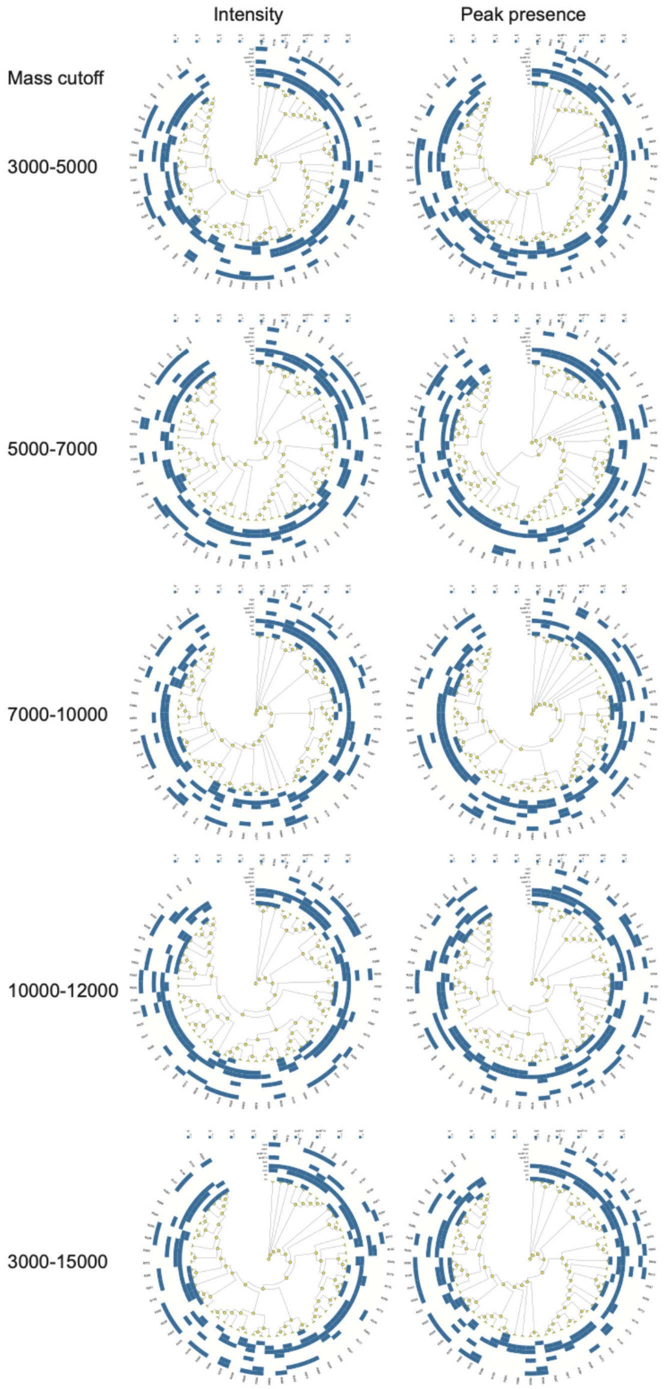

Figure S2. Dendrogram of 56 Escherichia coli isolates with associated extraintestinal virulence genes, generated based on protein mass fingerprints (PMFs) from MALDI-TOF MS with varying mass cutoffs and clustering parameters.

Table S1. Primers information.

Gene	Sequence (5'-3')	Amplification length (bp)	Annealing Temperature (℃)	Aim	Reference
E. coli 16S	GACCTCGGTTTAGTTCACAGA<br>CACACGCTGACGCTGACCA	585	55	Identification for *E. coli*	[1]
fliC	AGCTGCAACGGTAAGTGATTT<br>GGCAGCAAGCGGGTTGGTC	949	65	Intestinal virulence gene identification to exclude IEPC	[2]
stx1	TGTCGCATAGTGGAACCTCA<br>TGCGCACTGAGAAGAAGAGA	655	65		
stx2	CCATGACAACGGACAGCAGTT<br>TGTCGCCAGTTATCTGACATTC	477	65		
eae	CATTATGGAACGGCAGAGGT<br>ACGGATATCGAAGCCATTTG	375	65		
rfbE	CAGGTGAAGGTGGAATGGTTGTC<br>TTAGAATTGAGACCATCCAATAAG	296	65		
hlyA	ACGATGTGGTTTATTCTGGA<br>CTTCACGTGACCATACATAT	165	65		
SFA	CTCCGGAGAACTGGGTGCATCTTAC<br>CGGAGGAGTAATTACAAACCTGGCA	410	58	Extraintestinal virulence gene Identification to confirm ExPEC	[3]
ISS	GTGGCGAAAACTAGTAAAACAGC<br>CGCCTCGGGGTGGATAA	762	58		
TSH	GGTGGTGCACTGGAGTGG<br>AGTCCAGCGTGATAGTGG	642	58		
kpsMT II	GCGCATTTGCTGATACTGTTG<br>CATCCAGACGATAAGCATGAGCA	272	58		
kpsMT K1	TAGCAAACGTTCTATTGGTGC<br>CATCCAGACGATAAGCATGAGCA	153	58		
iucC	CGCCGTGGCTGGGGTAAG<br>CAGCCGGTTCACCAAGTATCACTG	541	58		
hlyD	CTCCGGTACGTGAAAAGGAC<br>GCCCTGATTACTGAAGCCTG	904	54		
papC	GACGGCTGTACTGCAGGGTGTGGC<br>ATATCCTTTCTGCAGGGATGCAATA	328	63		
ibeA	AGGCAGGTGTGCGCCGCGTAC<br>TGGTGCTCCGGCAAACCATGC	171	63		
sitA	AGGGGGCACAACTGATTCTCG<br>TACCGGGCCGTTTTCTGTGC	608	63		

Reference

1. Parvin, M.S.; Talukder, S.; Ali, M.Y.; Chowdhury, E.H.; Rahman, M.T.; Islam, M.T. Antimicrobial Resistance Pattern of Escherichia Coli Isolated from Frozen Chicken Meat in Bangladesh. *Pathogens* 2020, *9*.
2. Bai, J.; Shi, X.; Nagaraja, T.G. A Multiplex PCR Procedure for the Detection of Six Major

Virulence Genes in Escherichia Coli O157:H7. *J. Microbiol. Methods* **2010**, *82*, 85–89, doi:10.1016/j.mimet.2010.05.003.
3. Xia, X.; Meng, J.; Zhao, S.; Bodeis-Jones, S.; Gaines, S.A.; Ayers, S.L.; McDermott, P.F. Identification and Antimicrobial Resistance of Extraintestinal Pathogenic Escherichia Coli from Retail Meats. *J. Food Prot.* **2011**, *74*, 38–44, doi:10.4315/0362-028X.JFP-10-251.
